# Switching Adolescent High-Fat Diet to Adult Control Diet Restores Neurocognitive Alterations

**DOI:** 10.3389/fnbeh.2016.00225

**Published:** 2016-11-21

**Authors:** Chloé Boitard, Shauna L. Parkes, Amandine Cavaroc, Frédéric Tantot, Nathalie Castanon, Sophie Layé, Sophie Tronel, Gustavo Pacheco-Lopez, Etienne Coutureau, Guillaume Ferreira

**Affiliations:** ^1^Institut national de la Recherche Agronomique (INRA), Nutrition and Integrative Neurobiology, UMR 1286Bordeaux, France; ^2^Université de BordeauxBordeaux, France; ^3^Centre National de la Recherche Scientifique (CNRS), Institut de Neurosciences Cognitives et Intégratives d’Aquitaine, UMR 5287Bordeaux, France; ^4^Institut National de la Santé et de la Recherche Médicale (INSERM), U1215 Neuro Centre MagendieBordeaux, France; ^5^Biological and Health Sciences Division, Campus Lerma, Metropolitan Autonomous University (UAM)Lerma, Mexico

**Keywords:** hippocampus, amygdala, obesity, learning, adolescence, neurogenesis, rat

## Abstract

In addition to metabolic and cardiovascular disorders, obesity is associated with adverse cognitive and emotional outcomes. Its growing prevalence in adolescents is particularly alarming since this is a period of ongoing maturation for brain structures (including the hippocampus and amygdala) and for the hypothalamic-pituitary-adrenal (HPA) stress axis, which is required for cognitive and emotional processing. We recently demonstrated that adolescent, but not adult, high-fat diet (HF) exposure leads to impaired hippocampal function and enhanced amygdala function through HPA axis alteration (Boitard et al., 2012, 2014, 2015). Here, we assessed whether the effects of adolescent HF consumption on brain function are permanent or reversible. After adolescent exposure to HF, switching to a standard control diet restored levels of hippocampal neurogenesis and normalized enhanced HPA axis reactivity, amygdala activity and avoidance memory. Therefore, while the adolescent period is highly vulnerable to the deleterious effects of diet-induced obesity, adult exposure to a standard diet appears sufficient to reverse alterations of brain function.

## Introduction

Overconsumption of energy-dense, palatable foods is a recognized source of weight gain and obesity. Obesity is a serious public health challenge, causing physical disability and premature death as well as cognitive disturbances in adults (Nilsson and Nilsson, 2009; Sellbom and Gunstad, 2012; Francis and Stevenson, 2013) and adolescents (Cserjési et al., 2007; Li et al., 2008; Khan et al., 2015). While the incidence of overweight and obesity is increasing in all age ranges, it is particularly notable in adolescents (Ogden et al., 2012). This is alarming since adolescence is a period of neurobehavioral shaping required for life-long cognitive processing (Spear, 2000).

Adolescence is particularly sensitive to environmental challenges, like diet, and there is now compelling evidence that an energy-dense diet is more harmful when consumed during adolescence than in adulthood. The overconsumption of sugar or fat throughout adolescence, but not adulthood, is associated with changes in reward-related behaviors in rats including deficits in motivation (Vendruscolo et al., 2010) and attenuated conditioned place preference induced via a palatable food reward (Privitera et al., 2011). We and others have also demonstrated significant deleterious effects on hippocampal function specifically following adolescent consumption of a high-fat diet (HF) and/or high-sugar diet. Rodents that consume a diet supplemented with fat and/or sugar throughout adolescence, but not during adulthood, show impaired performance on a range of hippocampal-dependent behavioral tasks including object location memory (Valladolid-Acebes et al., 2013; Reichelt et al., 2015), spatial memory (Boitard et al., 2014; Hsu et al., 2015; Klein et al., 2016) and relational memory (Boitard et al., 2012). These cognitive deficits are associated with overexpression of hippocampal pro-inflammatory cytokines (Boitard et al., 2014; Hsu et al., 2015) and decreased levels of hippocampal neurogenesis (Boitard et al., 2012). Adolescent HF consumption (from weaning to adulthood) also leads to enhanced aversive memories and emotion-induced neuronal activation of the basolateral complex of the amygdala (BLA) as well as hypothalamo-pituitary-adrenal (HPA) axis deregulations. In contrast, these changes are not observed when the same diet is consumed in adulthood (Boitard et al., 2015). Taken together, these data illustrate that the adolescent period is especially sensitive to the effect of HF and high-sugar diets on memory.

There is now growing interest in determining whether the behavioral and neuronal modifications caused by an energy-dense adolescent diet are permanent. Therefore, in the present study, we investigated whether the neurobehavioral deficits induced by adolescent exposure to a HF could be reversed after removal of the HF. Rats were fed a HF immediately after weaning for 3 months and then were either maintained on the HF or switched to a control diet for an additional 3 months. At adulthood, the rats were tested on both hippocampal- (Morris Water Maze, MWM) and amygdala-dependent (conditioned odor avoidance, COA) memory tasks. A number of metabolic parameters were also measured. Critically, our results demonstrate that shifting to the control diet during adulthood was sufficient to reduce bodyweight and to partially restore all neurobehavioral and endocrine processes that were disrupted following adolescent HF consumption. This indicates that the detrimental neurocognitive effects associated with adolescent HF intake are reversible and can be either restored or maintained depending on the composition of adult diet.

## Materials and Methods

All experiments were conducted in agreement with the French (Directive 87-148, Ministère de l’Agriculture et de la Pêche) and international legislation (directive 2010-63, September 22, 2010, European Community) and were approved by the local ethical committee (agreement number 5012047-A).

### Animals and Diets

**Figure 1:**
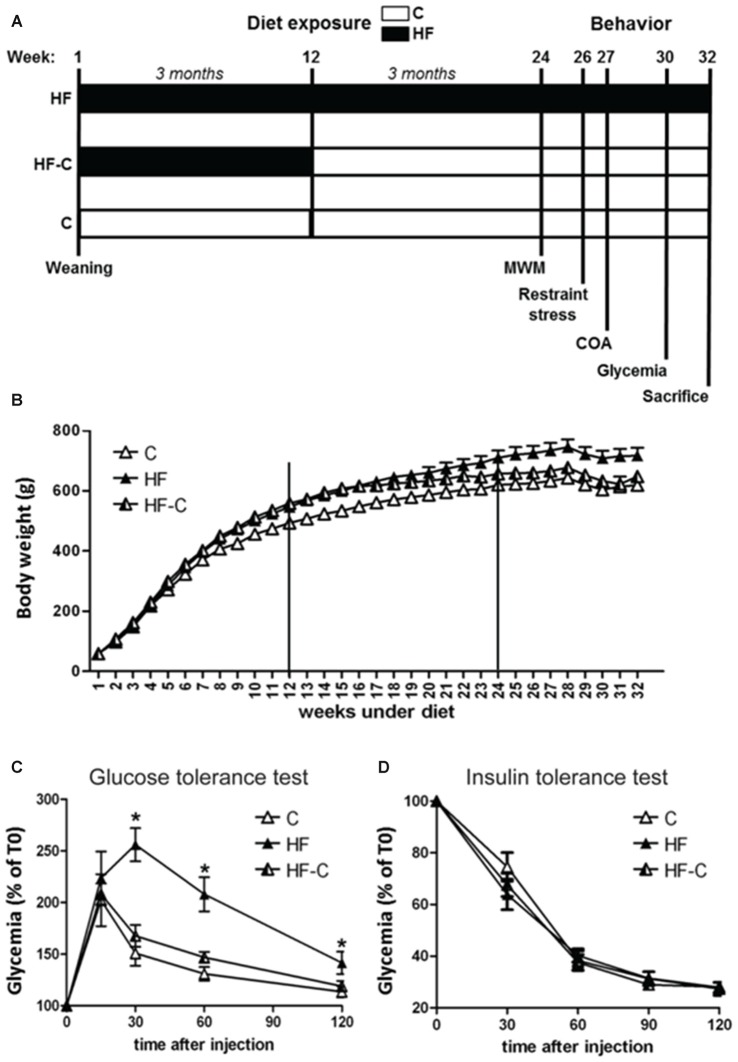
**(A)** Timeline of the experiments. Diet regimes began at weaning. Rats in group high-fat diet (HF) were continuously exposed to the HF (black bars) from weaning to sacrifice. Rats in group HF-C were given the HF from weaning for 12 weeks (black bars) and were then shifted to the control diet (white bars) until sacrifice. Rats in the control group (C) were given access to the control diet from weaning until sacrifice. Behavioral assessment began after 24 weeks on the diet regime and rats were sacrificed after 32 weeks. **(B)** Bodyweight for each group from weaning to sacrifice. Rats were weighed once per week. **(C)** Glucose metabolism was assessed 30 weeks after starting the diets. Glucose levels were higher in rats maintained on the HF (black triangles) 30–120 min after glucose injection. *p < 0.05 when compared to both C and HF-C groups. **(D)** Intra-peritoneal (i.p.) insulin tolerance test. Injection of insulin induced a similar decrease in blood sugar for all groups.

Animals were 50 Wistar naïve male rats (Janvier, France) aged 3 weeks old on arrival. They were housed in groups of 2–4 in polycarbonate cages (48 cm × 26 cm × 21 cm) in an air-conditioned (22 ± 1^°^C) colony room maintained under a 12:12 light/dark cycle. Rats had *ad libitum* access to food and water and were weighed once a week from arrival to sacrifice. On arrival, rats were divided into three groups with similar weights. The first group received only a control diet (C; *n* = 17) offering 2.9 Kcal/g (consisting of 60% carbohydrate, mostly from starch, and 2.5% fat (A04 SAFE, Augy, France); group C), the second group received only a HF (*n* = 15) offering 4.7 Kcal/g (consisting of 24% (45% kcal) fat, mostly saturated fat from lard, and 41% (35% kcal) carbohydrate, with 20% (17.5% kcal) from sucrose (D12451, Research Diets, New Brunswick, NJ, USA); group HF), and the third group received HF from arrival for a duration of 12 weeks, i.e., throughout adolescent development (from weaning to adulthood; see Spear, 2000), directly followed by exposure to the control diet at adulthood (group HF-C; *n* = 18, see Figure 1A). One week before the behavioral task, rats were isolated in individual cages (35 cm × 23 cm × 19 cm), to perform behavioral and metabolic assessments under optimal conditions, and habituated to handling by the experimenter. Behavioral experiments were performed on adult rats, starting after 6 months of diet exposure (see Figure 1A).

### Behavioral Tasks

#### Morris Water Maze (MWM)

##### Apparatus

A circular tank (150 cm in diameter and 50 cm high) was filled with opaque water (22 ± 2°C). A platform (10 cm diameter, 30 cm from the edge of the tank) was submerged 5 cm beneath the water surface and was not visible to the rats. Visual cues were provided on the walls of the room to allow spatial navigation. A camera wired to an automated tracking system (SMART v2.5.20, Panlab, Barcelona, Spain) recorded the rat’s pathway and behavior.

##### Learning schedule

The MWM protocol began after 24 weeks (6 months) of diet intervention (Figure 1A). As previously described (Boitard et al., 2014), rats were trained to locate the platform for five consecutive days. Before the first trial, rats were placed on the submerged platform for 30 s. Rats were given six trials per day, with different starting locations on each trial, following a pseudo-random sequence. Then, each trial consisted in a swim followed by a 30 s rest on the platform. Rats that did not reach the platform within 90 s were guided to it. The inter-trial interval was 15 s. Latency to reach the platform, distance traveled and swimming speed were recorded.

##### Memory assessment

Memory was assessed via probe tests occurring 2 h and 4 days (Long-term memory (LTM) assessment) after the final training session. The platform was removed and rats were allowed to navigate in the water maze for 90 s. Latency to reach the target annulus, time spent in the quadrants (each representing $\frac{1}{4}$ of the maze) and the number of each annulus crossings (imaginary circular zones, one in each quadrant, the target annulus being the one where the platform was located during learning) were recorded (Maei et al., 2009). Since our previous results indicate that HF mainly impacts the number of target annulus crossings (Boitard et al., 2014), we analyzed this parameter by comparing it to chance level (25%) using one sample *t*-tests. Rats failing to learn or accurately remember the location of platform during the test 2 h after training (threshold being less than 30 s to enter the target annulus) were removed from the analyses (final number of animals per group: C: 9/17, HF: 10/15 and HF-C: 11/18).

#### Conditioned Odor Avoidance (COA)

The COA protocol began after 27 weeks (6.5 months) of diet intervention for all 50 rats (Figure 1A), as previously described (Boitard et al., 2015). Rats were water deprived 24 h before the start of the protocol and acclimated to a water deprivation regimen. Access to tap water was provided in a graded bottle (with 0.5 mL accuracy) for 15 min/day for 3 days between 09:00 and 11:00 in the home cage. On the acquisition day (day 4), rats had 15 min access to almond-scented water, composed of 0.01% benzaldehyde (Sigma Aldrich) diluted in tap water. This almond solution was chosen because previous studies indicated that its processing is mediated by its odor properties, not by its taste properties (Rusiniak et al., 1979; Desgranges et al., 2009). Indeed, anosmic rats were unable to reliably detect 2% almond-scented water (Rusiniak et al., 1979), whereas they performed as well as control for taste detection (Slotnick et al., 1997) indicating that 0.01% almond-scented water is processed by odor, but not taste, cues. The percentage of almond-scented water consumption during acquisition in respect to water consumption the day before was used to measure the strength of neophobic response. After a 30 min delay, rats received an intra-peritoneal (i.p.) injection of the visceral malaise-inducing drug, lithium chloride (LiCl, 0.075 M, 0.75% of bodyweight, 25 mg/kg; Sigma Aldrich), in order to induce the COA. For the next 2 days (day 5 and 6), rats had access to odorless tap water for 15 min/day to re-establish baseline water intake. Finally, COA was assessed on day 7 by providing access to the almond-scented water for 15 min. The percentage of almond-scented water consumption during test in respect to almond-scented water consumption during acquisition was used to measure the strength of the avoidance.

### Metabolic Parameters

Glucose metabolism was assessed following the behavioral tasks, i.e., after a total of 30 weeks of diet exposure (Figure 1A). Glycemic level was measured in the morning after overnight fasting in all the rats and in response to either insulin or glucose i.p. injection (2UI/kg insulin for half of the rats and 20% glucose solution, 2 g/kg for the remaining rats). A blood drop provided by a tail nick allowed direct reading of the sugar blood level using Accu-Check^®^ devices (Roche diagnostics). Blood sampling from the same wound occurred at the time of injection (T0), and 30, 60, 90 and 120 min after injection for the insulin tolerance tests and T0, 15, 30, 60 and 120 min for the glucose tolerance test. For tolerance tests, data are reported as baseline (% of T0) in order to minimize inter-individual variation and then the total area under the curve (AUC) was calculated.

Triglycerides, cholesterol, leptin and insulin levels were measured in a subset of rats (9–15 rats/group) in plasma samples obtained from blood collected at sacrifice (32 weeks of diet exposure, Figure 1A) using specific kits (Cholesterol RTU, Triglycerides enzymatic PAP 150, Biomérieux, France) or milliplex (Rat serum adipokine kit, RADPK-81K, Millipore, Billerica, MA, USA) as previously described (Boitard et al., 2012, 2014).

### HPA Axis and Corticosterone Release

Corticosterone release in response to restraint stress was assessed 26 weeks (6.5 months) after the beginning of diet exposure in 7–8 rats/group (Figure 1A). Blood from each rat was quickly collected from a tail nick immediately before restraint stress (T0), directly after restraint stress (30 min after T0) and at two additional time points after the rat was returned to the home-cage (90 and 180 min after T0).

Corticosterone levels were also measured in a subset of rats (6–8 rats/group) after a systemic stressor (LiCl, 0.075 M, 25 mg/kg; Sigma Aldrich) used both to induce COA and to assess amygdala activity (see below). Blood sampling occurred 90 min after LiCl injection at the time of sacrifice (32 weeks of diet exposure, see Figure 1A).

Total corticosterone level was measured in plasma obtained from blood samples (centrifugation at 4000 rpm, 15 min, RT) by in-house radioimmunoassay (Richard et al., 2010). Briefly, after absolute ethanol steroid extraction from plasma samples, total corticosterone was measured by competition between cold corticosterone and radioactive corticosterone (3H) for a specific anti-corticosterone antibody provided by Dr. H. Vaudry (University of Rouen, France). The sensitivity of this technique is 0.3 μg/dl, with a 10% intra-assay and a 10% inter-assay variability. For the restraint stress-induced corticosterone response, the AUC was calculated in order to detect an overall effect and corticosterone levels.

### Amygdala Activation (c-Fos Labeling)

Injection of LiCl, used as the aversive stimulus in the COA, leads to gastric malaise and induces a strong activation of the central nucleus (CeA) and a moderate activation of the basolateral nucleus of the amygdala (BLA; Yamamoto et al., 1997; Koh et al., 2003; Ferreira et al., 2006). We evaluated CeA and BLA activation 90 min following LiCl injection using the neuronal activity marker, c-Fos.

Six to eight rats/group were injected with LiCl (0.075 M, 25 mg/kg; Sigma Aldrich) 90 min before being euthanized with an i.p. injection of a lethal dose of pentobarbital sodium (Ceva Santé Animale, France). Blood was collected transcardially (in order to measure plasma corticosterone, see above) before perfusion with 0.1 M phosphate buffered saline (PBS, pH = 7.4) followed by 4% paraformaldehyde (PFA) in PBS. Brains were quickly removed and stored at 4°C in a 4% PFA for 24 h to allow post-fixation. Plasma obtained from blood samples (centrifugation at 4000 rpm, 4°C for 15 min) was kept at −80°C before assessing metabolic parameters. The next day, brains were submerged in a 30% sucrose solution at 4°C for 48 h to allow cryoprotection before being frozen in isopentane and stored at −80°C. Brains were sectioned coronally at 40 μm using a cryostat (Leica, Paris, France), and stored in a cryoprotective ethylene-glycol solution at −20°C. Coronal sections from 2.5 mm to 3.2 mm posterior to bregma, sampling the whole amygdala (Paxinos and Watson, 2013), were treated. Free-floating sections were rinsed extensively (0.1 M PBS 3× 10 min) and incubated in 3% bovine serum albumin (BSA) and 0.3% Triton (PBS-BSA-T) to block nonspecific binding sites and to facilitate antibody penetration. Sections were first incubated with the primary anti-c-Fos antibody (Santa Cruz Biotechnology, anti-c-Fos rabbit polyclonal antibody, 1:1000 diluted in PBS-BSA-T, 18 h, RT) and incubated with hydrogen peroxide (H_2_O_2_, 30 min) to eliminate endogenous peroxidase activity. Sections were then incubated with the biotinylated secondary antibody (Vector Laboratories, goat anti-rabbit IgG, diluted 1:1000 in PBS, 2 h, RT) followed by incubation in the avidin–biotin-peroxidase complex solution (ABC solution; Vectastain, Vector laboratories, diluted 1:1000 in PBS, 1 h, RT). Sections were rinsed in sodium acetate (2× 10 min), and the peroxidase complex was visualized after incubation for 30 min in a mix containing diaminobenzidine, sodium acetate, glucose and glucose oxidase leading to a black precipitate. Between each treatment, sections were thoroughly rinsed with 0.1 M PBS (3× 10 min). Finally, sections were mounted on gelatin-coated slides, air-dried, dehydrated in an ascending alcohol series, cleared in xylene and cover slipped.

Labeling was quantified bilaterally on four sections spaced 240 μm apart, chosen to cover the whole amygdala (2.5–3.4 mm posterior to bregma) and representing the same four levels for all rats. Each section of interest was photographed using Nikon-ACT-1^®^ software and labeled cells were counted independently in the CeA and BLA nuclei with ImageJ^®^ software on a surface representing 0.85 mm^2^. Results are expressed for a surface of 1 mm^2^.

### Hippocampal Neurogenesis (DCX Labeling)

Neurogenesis was evaluated by determining the number of immature neurons in the dentate gyrus, characterized by the endogenous marker doublecortin (DCX), a cytoplasmic protein expressed transiently in newborn neurons only (Brown et al., 2003).

In the subset of rats (6–8 rats/group), coronal sections (40 μm) from 2.8 mm to 4.4 mm posterior to bregma, sampling the whole hippocampus (according to Paxinos and Watson, 2013), were treated. Free-floating sections were rinsed (PBS 3× 10 min, 0.1 M) and incubated with methanol/hydrogen peroxide (0.5%) followed by 3% serum normal rabbit and 0.3% Triton (PBS-SNr-T) to block nonspecific binding sites and to facilitate antibody penetration. Sections were incubated with the primary anti-DCX antibody (Santa Cruz Biotechnology, anti-DCX goat polyclonal antibody, 1:1000 diluted in PBS-SNr-T, 72 h, 4°C) followed by biotinylated secondary antibody (Dako, rabbit anti-goat IgG, diluted 1:200 in PBS, 1.5 h, RT) and then incubated in the avidin–biotin-peroxidase complex solution (ABC solution; Vectastain, Vector laboratories, diluted 1:200 in PBS, 1.5 h, RT). Sections were rinsed in sodium acetate (2× 10 min), and the peroxidase complex was visualized after incubation for 30 min in a mix containing diaminobenzidine, sodium acetate, glucose and glucose oxidase. Between each treatment, sections were rinsed with 0.1 M PBS (3× 10 min). Finally, sections were mounted on gelatin-coated slides, air-dried, dehydrated in an ascending alcohol series, cleared in xylene and cover slipped.

Labeling was quantified bilaterally on four sections spaced 400 μm apart, chosen to cover the whole dentate gyrus (2.8–4.4 mm posterior to bregma) and representing the same four levels for all rats. Each section of interest was photographed using Nikon-ACT-1^®^ software and labeled cells were counted with ImageJ^®^ software.

### Data Analysis

Data are expressed as the mean ± SEM. All analyses were conducted using Statview software and statistical significance was set at *p* < 0.05. Outliers (individual results significantly differing from the group mean) were calculated with GraphpadQuickCalcs online application, using the extreme studentized deviate method, and were removed from corresponding analyses. The diet effect was assessed using ANOVAs followed by Fisher’s *post hoc* tests when ANOVAs were significant. For the MWM, the number of target annulus crossings for each group was compared to chance level (25%) using one sample *t*-tests.

## Results

### Bodyweight Across Diet Exposure

There was no difference in bodyweight between the groups before starting the diet exposure (*F*_(2,47)_ < 1, *p* = 0.90, see Table 1). An analysis restricted to the first 3 months of diet exposure (week 1–12) found a significant effect of diet (*F*_(2,47)_ = 11.14, *p* < 0.001), time (*F*_(11,517)_ = 4806.56, *p* < 0.001) and an interaction between these factors (*F*_(22,517)_ = 9.16, *p* < 0.001, see Figure 1B), indicating that the rate of weight gain differed between the groups in the first 3 months. *Post hoc* analyses indicated that the HF fed rats (groups HF and HF-C) were significantly heavier than C fed rats (Fisher’s *post hoc*: *p* < 0.001) but no significant difference was found between the two HF fed groups (*p* = 0.26). At the 12th week, rats under HF were significantly heavier than C fed rats (*F*_(2,47)_ = 12.3, *p* < 0.001; *post hoc*: *p* < 0.001; 11–13% overweight compared to group C). At this time point, the bodyweight of HF and HF-C groups did not differ (Fisher’s *post hoc*: *p* = 0.41) and rats in group HF-C group were shifted to the control diet.

**Table 1:** **Mean body weight and metabolic measures (± SEM)**.

	C	HF	HF-C
Initial body weight	58.18 (0.48)	58.13 (1.01)	57.94 (0.89)
Body weight after 12 weeks	493.35 (6.6)	546.67 (12.41)*	558.56 (10.64)*
Body weight after 24 weeks	619.00 (9.64)	709.93 (23.57)*°	656.44 (15.27)*
Body weight after 32 weeks	618.67 (13.32)	716.57 (28.27)*°	647.61 (14.08)
After 32 weeks
Triglycerides (g/l)	124.23 (17.08)	95.226 (17.6)	13212 (11.0)
Cholesterol (g/l)	80.06 (4.82)	96.14 (6.41)*°	70.03 (3.85)
Leptin (ng/ml)	13.57 (2.93)	29.13 (4.62)*	19.02 (4.83)
Insulin (ng/ml)	3.79 (0.65)	4.51 (0.31)°	2.67 (0.53)
Glucose (mg/dl)	96.82 (1.62)	96.67 (1.8)	99.5 (2.26)

*Significantly different from group C; °significantly different from group HF-C.

For week 13–24 (3–6 months), a significant effect was detected for diet (*F*_(2,47)_ = 8.16, *p* < 0.001), time (*F*_(11,517)_ = 316.62, *p* < 0.001) and the diet × time interaction (*F*_(22,517)_ = 10.1, *p* < 0.001). Specifically, rats in group HF and HF-C were significantly heavier than group C (HF vs. C: *p* < 0.001;HF-C vs. C: *p* = 0.005) and groups HF and HF-C did not differ (*p* = 0.31) despite cessation of the HF in group HF-C. At the 24th week, when behavioral testing started, an overall diet effect was detected (*F*_(2,47)_ = 7.29, *p* = 0.002). Rats maintained under HF were significantly heavier than group C (Fisher’s *post hoc*: *p* < 0.001, 15% overweight) and group HF-C (*p* = 0.03), which did not differ from each other (*p* = 0.11).

Finally, from week 25–32 (6–8 months), there was a significant effect of diet (*F*_(2,47)_ = 8.81, *p* < 0.001) and time (*F*_(7,329)_ = 18.3, *p* < 0.001) but no significant interaction between these factors (*F*_(14,329)_ = 1.06, *p* = 0.393), indicating a similar rate of weight gain in all groups. Rats maintained under the HF were overweight compared to both group C (Fisher’s *post hoc*: *p* < 0.001) and group HF-C (*p* = 0.005). At the 32th week (the time of sacrifice), and similar to the 24th week (beginning of behavioral assessment), a diet effect was detected (*F*_(2,47)_ = 7.70, *p* = 0.001). *Post hoc* analyses revealed that animals maintained under HF diet were significantly heavier than C rats (*p* < 0.001, 16% overweight) and HF-C rats (*p* = 0.009), and HF-C rats did not differ from C rats (*p* = 0.24).

### Metabolic Changes with Diet Exposure

Glucose metabolism was assessed 30 weeks after starting the diets. Glycemic level was not affected by the diets after overnight fasting (*F*_(2,47)_ < 1, *p* = 0.504). However, glucose i.p. injection resulted in a higher and protracted blood sugar level increase in the HF group compared to both C and HF-C groups (diet effect: *F*_(2,17)_ = 7.64, *p* < 0.005, time effect: *F*_(4,68)_ = 77.69, *p* < 0.001, diet × time interaction: *F*_(8,68)_ = 5.76, *p* < 0.001, AUC: *F*_(2,17)_ = 7.65, *p* < 0.005; Figure 1C). Blood glucose levels were higher in the HF group compared to both C and HF-C groups 30, 60 and 120 min after injection (Fisher’s *post hoc*: all *p* values < 0.03). Groups HF-C and C did not differ at any time point (all *p* values > 0.1). Thus, glucose homeostasis was affected following long-term HF exposure, but HF exposure alone was not sufficient to cause enduring changes. In contrast, insulin i.p. injection induced a similar decrease in blood sugar level for all groups (diet effect: *F*_(2,21)_ = 0.26, *p* = 0.78, time effect: *F*_(4,84)_ = 417.32, *p* < 0.001, diet × time interaction: *F*_(8,84)_ = 1, *p* = 0.45, AUC: *F*_(2,21)_ = 1.21, *p* < 0.32; Figure 1D), showing HF exposure did not induce insulin resistance under our conditions.

Other metabolic parameters were measured in the plasma at the time of sacrifice (see Table 1). Triglyceride levels were not affected by the diet (*F*_(2,33)_ = 2, *p* = 0.25) however, cholesterol levels differed between groups (*F*_(2,37)_ = 7.0, *p* < 0.003). Specifically, HF group had higher cholesterol levels compared to group C (*p* = 0.035) and group HF-C (*p* < 0.001), which did not differ from each other (*p* = 0.13). Leptin levels marginally differed between groups (*F*_(2,31)_ = 3.1, *p* = 0.06). *Post hoc* analyses indicated higher levels in the HF group compared to group C (*p* = 0.02) but no other comparisons were significant (*p* values < 0.1). Insulin levels were also marginally affected by the diet (*F*_(2,29)_ = 3.0, *p* = 0.07) with lower insulin levels observed in group HF-C compared to group HF (Fisher’s *post hoc*: *p* = 0.03).

### Spatial Hippocampal-Dependent Memory and Hippocampal Neurogenesis

**Figure 2:**
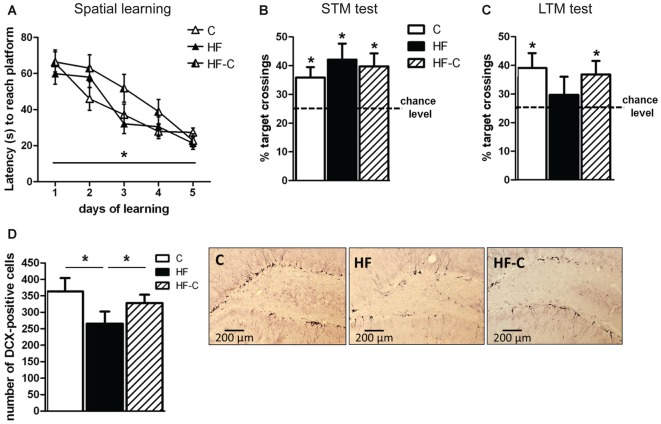
**Spatial hippocampal-dependent memory and hippocampal neurogenesis. (A)** Performance in the Morris Water Maze (MWM) across training trials. All groups learned the location of the platform and showed a decreased latency to reach the platform across days. *p < 0.05 (repeated measure’s ANOVA: time effect). **(B)** Short-term memory (STM) was assessed 2 h after the final training trial. The percentage of target annulus crossing was significantly greater than chance level (25%) for all groups. *p < 0.05 (one-sample *t*-test). **(C)** Long-term memory (LTM) was assessed 4 days after the final training session. The percentage of target annulus crossings was significantly greater than chance for groups C (white bar) and HF-C (stripped bar) but not for group HF (black bar). *p < 0.05 (one-sample *t*-test). **(D)** Less doublecortin (DCX)-positive cells were observed in the dentate gyrus of group HF than in groups C and HF-C. *p < 0.05 when compared to both C and HF-C groups (significant one-way ANOVA followed by Fisher’s *post hoc*). Representative photomicrographs of DCX-immunoreactivity in the dentate gyrus of groups C, HF and HF-C.

Spatial learning was assessed in the MWM. All groups learned the location of the hidden platform during the 5 days of training (six trials per day), as evidenced by a decreased latency to reach the platform across days (session effect: *F*_(4,108)_ = 33.3, *p* < 0.001; diet effect and interaction: *F* values < 2, *p* values > 0.1; Figure 2A). Similar results were obtained for the distance traveled and no group difference was found in swimming speed (data not shown).

A first probe test was performed 2 h after the last training trial in order to evaluate learning performance. Only rats able to accurately remember the platform location during this probe test (i.e., entering the target annulus in less than 30 s) were kept for LTM assessment. As shown in Figure 2B, all groups showed a preference for the target annulus compared to the other annuli (comparison to 25% chance level using one sample *t*-test: *t*_(8)_ = 3.0, *t*_(9)_ = 3.0, and *t*_(10)_ = 3.3, *p* < 0.02 for groups C, HF and HF-C respectively, no diet effect: *F*_(2,27)_ < 1). The number of annuli crossings was similar in all groups (10.1 ± 1.8, 10.8 ± 2.8 and 9.1 ± 2.1 for C, HF and HF-C, respectively; *F*_(2,27)_ < 2, *p* > 0.1).

During the LTM test (4 days after final training session), groups C and HF-C still exhibited significantly more crossings in the target annulus compared to 25% chance level (*t*_(8)_ = 2.7, *p* = 0.03; *t*_(10)_ = 2.4, *p* < 0.04 for C and HF-C, respectively) whereas group HF did not cross the target annulus more than in a random navigation (*t*_(9)_ < 1, *p* < 0.1, Figure 2C). However, no diet effect was revealed for target annulus crossings (*F*_(2,27)_ < 1, *p* > 0.1) indicating that the groups did not differ from each other. Moreover, the number of annuli crossings was not different between groups (8.8 ± 2.4, 7.4 ± 3.2 and 7.5 ± 4.5 for groups C, HF and HF-C, respectively; *F*_(2,27)_ < 1, *p* > 0.1).

Hippocampal neurogenesis was assessed by counting DCX-positive cells in the dentate gyrus of the hippocampus 7 weeks after the end of MWM to avoid any potential effect of spatial learning on neurogenesis (e.g., Tronel et al., 2010). A diet effect was revealed (*F*_(2,18)_ = 4.1, *p* = 0.035, Figure 2D) such that the HF group showed significantly less DCX-positive cells compared to groups C (Fisher’s *post hoc*: *p* = 0.012) and HF-C (*p* = 0.046). In addition, DCX-positive cell numbers in C and HF-C groups did not differ from each other (*p* = 0.40).

### Avoidance Amygdala-Dependent Memory and Amygdala Activation

**Figure 3:**
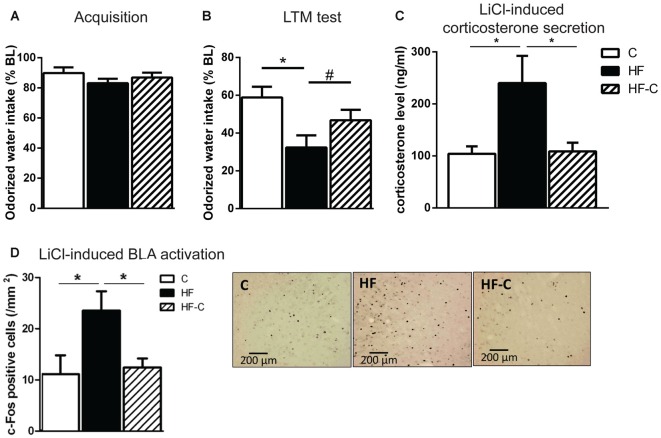
**Aversive amygdala-dependent memory and amygdala activation. (A)** Odorized water intake did not differ between groups on acquisition day. **(B)** Aversive memory was assessed 3 days after the acquisition trial. All groups showed an avoidance of the odorized water however, the strength of the avoidance was greater in group HF (black bar). *p < 0.01, ^#^*p* = 0.09 (significant one-way ANOVA followed by Fisher’s *post hoc*). **(C)** Rats in the group HF showed increase circulating corticosterone levels 90 min after lithium chloride (LiCl) injection compared to groups C (white bar) and HF-C (striped bar). *p < 0.05 when compared to both C and HF-C groups (significant one-way ANOVA followed by Fisher’s *post hoc*). **(D)** Rats in group HF showed a higher number of c-Fos positive cells in the basolateral amygdala (BLA) 90 min after LiCl injection than groups C and HF-C. *p < 0.05 (significant one-way ANOVA followed by Fisher’s *post hoc*). Representative photomicrographs of c-Fos immunoreactivity in the BLA for groups C, HF and HF-C.

Avoidance memory was assessed via COA. To avoid inter-individual differences in liquid consumption, almond-scented odor consumption during acquisition was expressed as a percentage of water consumption the day before. On the acquisition day, all groups showed reduced odorized water intake with respect to water consumption the day before (paired *t*-test: *t*_(16)_ = 3.0, *t*_(14)_ = 5.2 and *t*_(17)_ = 3.8 for groups C, HF and HF-C, respectively, *p* values < 0.01) indicating a slight yet similar neophobic response for all groups (83%–89% of water consumption, *F*_(2,47)_ < 1; Figure 3A). Gastric malaise was induced 30 min after consumption by an i.p. LiCl injection. During the next 2 days, all groups showed similar, normal water consumption (95%–105% of water baseline, *F*_(2,47)_ < 1). On the next day, avoidance memory was assessed and almond-scented odor consumption during test was expressed as a percentage of odorized water consumption during acquisition. All groups showed reduced odorized water intake with respect to acquisition (paired *t*-test: *t*_(16)_ = 6.7, *t*_(14)_ = 10.4 and *t*_(17)_ = 9.3 for groups C, HF and HF-C, respectively, *p* values < 0.001) indicating an avoidance of the odorized water. However, there was a significant effect of diet on the strength of the avoidance (*F*_(2,47)_ = 4.9, *p* = 0.012; Figure 3B). Specifically, the HF group showed a significantly stronger avoidance than group C (Fisher’s *post hoc*: *p* = 0.003) and a tendency towards a stronger avoidance than group HF-C (*p* = 0.09). In addition, C and HF-C group did not differ from each other (*p* = 0.14).

Our previous results demonstrate that exposure to HF for 3 months induces higher plasma corticosterone release and c-Fos-positive cells in the BLA 90 min after LiCl injection (Boitard et al., 2015). Here, we assessed if C consumption after exposure to HF for 3 months could restore these endocrine and neurobiological responses. A clear diet effect was revealed on corticosterone levels (*F*_(2,18)_ = 6.5; *p* = 0.007; Figure 3C). The HF group differed from groups C (*p* = 0.004) and HF-C (*p* = 0.007) which did not differ from each other (*p* = 0.90). Regarding c-Fos expression in the amygdala, no diet effect was found in the CeA (mean ± SEM number of c-Fos positive cells: C: 42.4 ± 6.2, HF: 58.3 ± 14.4, HF-C: 68.1 ± 5.5; *F*_(2,19)_ = 1.9; *p* = 0.17; data not shown), as previously reported (Boitard et al., 2015). However, a clear diet effect was detected in the BLA (*F*_(2,20)_ = 4.0; *p* = 0.034; Figure 3D) revealing increased neuronal c-Fos expression in the HF group compared to both C (*p* = 0.028) and HF-C groups (*p* = 0.018), which did not differ from each other (*p* = 0.95).

### Corticosterone Release

**Figure 4:**
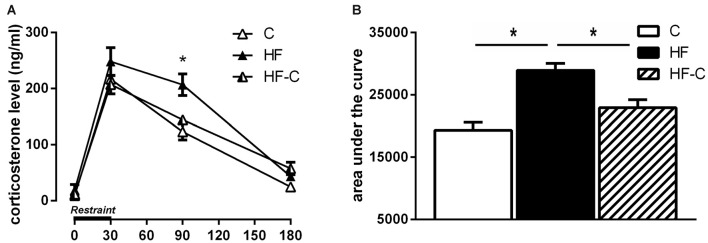
**Corticosterone secretion following restraint stress. (A)** Higher corticosterone levels were found in the group HF (black bar) 90 min after restraint stress compared to group C (white bar) and group HF-C (striped bar). *p < 0.05 when compared to both C and HF-C groups. **(B)** The area under the curve (AUC) was significantly higher for the HF group compared with groups C and HF-C. *p < 0.05 (significant one-way ANOVA followed by Fisher’s *post hoc*).

To better characterize the effect of HF intake on corticosterone response, we compared the time course of plasma corticosterone levels in the three groups following restraint stress. A repeated-measures ANOVA indicated a significant effect of diet (*F*_(2,20)_ = 9.46, *p* = 0.0013), time (*F*_(3,60)_ = 193.31, *p* < 0.0001) and a diet × time interaction (*F*_(6,60)_ = 2.89, *p* = 0.015; Figure 4A). As previously demonstrated (Boitard et al., 2015), no diet effect was revealed on basal corticosterone release (*F*_(2,21)_ = 0.95, *p* = 0.40) or 30 min after stress onset (*F*_(2,21)_ = 1.54, *p* = 0.24). However, a diet effect appeared 90 min after stress onset (*F*_(2,21)_ = 8.87, *p* = 0.002), with the HF group differing from both group C (*p* < 0.001) and group HF-C (*p* = 0.006) and no difference was found between groups HF-C and C (*p* = 0.25). This HF-induced protracted corticosterone release is temporary as, 180 min after stress induction, corticosterone levels did not differ between HF and the two other groups (*F*_(2,21)_ = 4.44, *p* = 0.024; Fisher’s *post hoc*: HF vs. C: *p* = 0.15; HF vs. HF-C: *p* = 0.26; HF-C vs. C: *p* = 0.007).

The AUC was significantly higher for the HF group compared to groups C and HF-C (*F*_(2,21)_ = 12.6; *p* < 0.001; Fisher’s *post hoc*: HF vs. C: *p* < 0.001; HF vs. HF-C: *p* = 0.005; HF-C vs. C: *p* = 0.048; Figure 4B). These data indicate a protracted corticosterone release in response to restraint stress in the HF group that is restored by C exposure after exposure to HF.

## Discussion

The adolescent period is particularly vulnerable to the detrimental cognitive effects of a diet laden with sugar or fat (Vendruscolo et al., 2010; Privitera et al., 2011; Boitard et al., 2012, 2014, 2015; Valladolid-Acebes et al., 2013; Hsu et al., 2015; Reichelt et al., 2015; Klein et al., 2016; Naneix et al., 2016). However, the present study, in conjunction with our previous studies, indicates that the neurocognitive alterations associated with adolescent HF intake are partially reversible. That is, 3 months after removal of the HF, rats with a history of adolescent HF consumption showed no deficits in amygdala-dependent memory and marginal improvements in hippocampal-dependent memory. Moreover, these rats did not show any enduring changes in weight, metabolism (cholesterol, leptin and glucose), corticosterone release, hippocampal neurogenesis or amygdala reactivity.

Contrary to our previous findings (Boitard et al., 2014), we failed to observe group differences in performance during the LTM test of the MWM. This may be due to the large number of rats excluded from all groups due to an inability to learn the task. Nevertheless, when the number of target annulus crossing was compared to chance level for each group, it was revealed that control-fed rats and rats switched from a HF to a control diet spent significantly more time in the target annulus compared to chance whereas rats maintained on a HF did not. Moreover, rats given a HF from weaning to sacrifice showed decreased levels of neurogenesis in the dentate gyrus as we previously reported in mice (Boitard et al., 2012). However, those that received adolescent HF (from weaning for 3 months) and were then switched to a standard control diet showed levels of hippocampal neurogenesis similar to rats with no history of HF consumption.

It was previously reported that adolescent intake of a HF led to long-lasting effects on hippocampal function. Mice consuming a HF starting during adolescence showed impaired object location memory, increased dendritic spine density and desensitization of leptin receptors in hippocampus (Valladolid-Acebes et al., 2013). Critically, these neurobehavioral modifications persisted despite a period (5 weeks) of restricted HF intake. However, not all diet-induced changes were enduring as, similar to the current study, leptin levels normalized following HF restriction (Valladolid-Acebes et al., 2013).

Taken together, these data indicate that reversal of adolescent HF effects may require abstinence from the diet rather than restricted intake only. Indeed, adult rats previously fed with HF then given a short-term (4 weeks) dietary reversal to standard control diet completely recovered hippocampal memory function (Sobesky et al., 2014). Similarly, a recent report indicates that mice maintained on HF for 3 months before being switched to low-fat diet for 2 months showed normalized hippocampal plasticity and hippocampal-dependent memory (Hao et al., 2016). Moreover, it should also be noted that, in our study, group HF-C did not differ in bodyweight from rats fed a control diet whereas, in the study by Valladolid-Acebes et al. (2013), mice remained significantly heavier than control fed mice at the time of behavioral assessment and sacrifice. It could therefore be argued that we may have observed cognitive deficits in the rats switched to a standard control diet if behavioral testing occurred when these rats were still overweight. However, this seems unlikely given that adult rats previously fed with a HF then switched to standard control diet for 4 weeks still weighed more than control fed animals (and not significantly less than the HF rats) but completely recovered hippocampal memory function (Sobesky et al., 2014).

Consistent with our previous findings obtained after 3 months of HF exposure starting at weaning (Boitard et al., 2015), we observed enhanced avoidance memory and amygdala reactivity as well as protracted corticosterone release after maintained HF exposure for 8 months (starting at weaning). While this data is purely correlative, we have previously shown that blockade of glucocorticoid receptors in the BLA of HF-fed rats normalizes aversive memory indicating a causal link between changes in amygdala modulation by glucocorticoids and an increase in emotional memory (Boitard et al., 2015). In contrast, 3 months of HF exposure starting at weaning was without effect 3 months after removal of the HF. That is, after 3 months of control diet, rats with a history of adolescent HF showed normal avoidance memory and neuronal activity in the basolateral amygdala. Corticosterone responses to both restraint and systemic stressors also did not differ from rats fed a control diet only.

Others have also reported that behavioral changes in non-hippocampal systems, caused by adolescent HF, can be rescued following diet cessation. Indeed, alterations in sucrose preference and dopamine system function observed after 12 weeks of adolescent HF were partially reversed 1 month after removal of the diet (Carlin et al., 2016). Similarly, rats exposed to a HF and high sugar diet for 10 days (postnatal 22–32) showed a reduction in sucrose preference when tested immediately after diet exposure. Rats were then shifted to a standard control diet until adulthood. At adulthood, sucrose preference was restored (Rabasa et al., 2016). However, sucrose preference was also restored at adulthood if rats were maintained on the HF-high sugar diet, thus it was not removal of the high energy diet *per se* that was responsible for the restoration of sucrose preference. In the present study, we showed that rats maintained on HF from weaning until behavioral testing at adulthood showed clear memory, neurobiological and endocrine changes. Switching from a HF to a control diet was therefore critical in reversing these alterations.

While the present results indicate that adolescent HF intake does not produce enduring neurocognitive modifications, it should be noted that animals were only tested during adulthood. It is possible that the effects of adolescent HF may resurface during another vulnerable period, such as aging. Indeed, the effects of HF intake are exacerbated in the aging brain (Ledreux et al., 2016). Aged rats that consumed a HF exhibited reduced hippocampal morphology and greater memory deficits compared to young HF fed rats and age-matched control fed rats (Ledreux et al., 2016). Moreover, a recent article indicates that early-life exposure to HF has long-term deleterious consequences (Wang et al., 2015). Exposure to HF for 4 months (starting at early adulthood) followed by 15 months of normal low-fat diet induces epigenetic modifications and synaptic dysfunction in the hippocampus as well as deficits in hippocampal-dependent memory despite restoration of normal weight and metabolic homeostasis (Wang et al., 2015). Therefore, while we observed that switching to a control diet after adolescent HF intake is sufficient to ameliorate neurocognitive deficits at adulthood, enduring deficits may be observed later in life or during demanding cognitive situations.

We previously demonstrated that 3 months of HF consumption from weaning to adulthood induced a range of neurocognitive alterations including reduced hippocampal neurogenesis (Boitard et al., 2012, 2014) as well as protracted corticosterone release and enhanced amygdala plasticity and aversive memory (Boitard et al., 2015). Here, we have shown that introducing a standard control diet for 3 months after adolescent HF consumption is sufficient to reverse these alterations. That is, the adverse effects of adolescent HF exposure can be overcome by restoring a proper nutritional diet at adulthood. Our results suggest that by introducing diet and weight management in adults, we can improve some of the negative effects of early life obesity.

## Author Contributions

CB and GF designed research; CB, AC and FT performed research; CB, SLP and GF analyzed data; GF supervised research; CB, SLP and GF wrote the manuscript. All authors edited the manuscript.

## Conflict of Interest Statement

The authors declare that the research was conducted in the absence of any commercial or financial relationships that could be construed as a potential conflict of interest.
